# Supplementary material for: fastBMA: scalable network inference and transitive reduction
Source: Gigascience. 2017 Aug 22;6(10):1–10. doi: 10.1093/gigascience/gix078 (PMC5632288; doi:10.1093/gigascience/gix078)
Supplement: Reviewer-1_Original-Submission-(Attachment).pdf [file gix078_Reviewer-1_Original-Submission-(Attachment).pdf]

The authors of the manuscript titled: “fastBMA: Scalable Network Inference and Transitive Reduction” have developed a fast and scalable gene regulatory network reconstruction algorithm which is a faster and more accurate version of their previous algorithm scanBMA. It also features a network post-processing method based on transitive reduction of graphs. Below are my comments on this manuscript.

In general the manuscript is relevant to current research, especially in the field of systems biology and biostatistics. It is well written and clearly understandable. It is a welcome addition to the arsenal of scalable algorithms for gene regulatory network inference. However, I think the paper can be improved significantly by addressing the following comments.

1) The authors claim that the transitive reduction based network post-processing method is a novel and important feature of their algorithm. Firstly, very similar techniques were previously used in many papers, some of which were cited by the authors in their manuscript. Therefore, I do not think it is appropriate to call it novel. Secondly, in the benchmarking studies, the transitive reduction method did not seem to improve the accuracy of the networks inferred by the fastBMA algorithm. If it does not improve the performance of fastBMA then why is it being packaged together with fastBMA and being presented as an important feature of the fastBMA algorithm?

2) In the “Background” section (under “Findings”) the authors cited many relevant research papers. However, in the regression based methods category the authors mostly cited their own work. I thinks the authors should cite other similar works in the same category, e.g. doi:10.1038/srep37140, http://dx.doi.org/10.1039/C4MB00053F,https://doi.org/10.1093/bioinformatics/bti487 .

3) It seems that the underlying principles of the fastBMA algorithm is written under the heading “Related work”. This is confusing since “related work” typically refers to similar work by other researchers.

4) The authors claimed that their algorithm can incorporate prior knowledge of the network topology in the inference process. In the benchmarking studies they have shown how prior knowledge improve the performance of their algorithm. However, I did not find a description of how prior knowledge is incorporated in the core algorithm. A brief description of this process will help readers understand the algorithm in its entirety.

5) The benchmarking studies performed in this manuscript are not convincing. The authors did not compare the performance of their algorithm with some of the most well known methods such as GENIE3 (http://dx.doi.org/10.1371/journal.pone.0012776 and JUMP3), JUMP3 (10.1093/bioinformatics/btu863) which were shown to be significantly superior to algorithms such as ARACNE, MRNET, CLR etc. which were used to compare the performance of scanBMA whose performance was compared with the fastBMA algorithm in this manuscript. To gain a better understanding of where their algorithm stands in terms of accuracy, compared to the current state of the art, they should compare the performances of their algorithm with the current top performers.

6) The authors did not properly discuss the weaknesses of their algorithm, for instance in which scenarios their algorithm is not expected to perform well?
