# Supplementary material for: fastBMA: scalable network inference and transitive reduction
Source: Gigascience. 2017 Aug 22;6(10):1–10. doi: 10.1093/gigascience/gix078 (PMC5632288; doi:10.1093/gigascience/gix078)
Supplement: Supplemental materials [file gix078_Supp.pdf]

Supplemental materials

Algorithm pseudocode

1.1 Model generation

```
INPUT: oddsRatio, hashLUT #hash value lookup table
Initialize(activeModels,keepModels,nextModels,evaluatedModels)
Initialize(bitTable[64][65326])
bestConfidence=0
While(activeModels != {} )
 nextModels.clear()
 ForEach model in activeModels #Add a new variable
  ForEach index in variableIndexList
   If(!model.contains(variable))
    If(bitTable.set(model.hash^hashLUT[variable], model.size()+1)){
     newModel=CholeskyInsert(model,variable)
     If(newModel.score < bestscore + 2*log(oddsRatio)
      nextModels.add(newModel)
      If(newModel.confidence < bestConfidence)
       bestConfidence=(newModel.confidence
      EndIf
     Endif;
    EndIf
   EndIf
  EndForeach
  ForEach index in model.variableList #Delete a variable
   If(bitTable(model.hash^hashLUT[index],model.size()-1)
    newModel=CholeskyDelete(model,variable)
    If(newModel.score < bestscore + 2*log(oddsRatio)
     nextModels.add(newModel)
     If(newModel.confidence < bestConfidence)
     bestConfidence=(newModel.confidence)
    EndIf
   EndIf
  EndForeach
 EndForeach
 keepModels.deletePoorModels()
 activeModels.deletePoorModels()
 keepModels.addModels(activeModels)
 nextModels.deletePoorModels()
 activeModels=nextModels;
EndWhile
```

© Oxford University Press 2005

1.2 Bit table filter

```
Function bitTable.set (hashValue, modelSize){
 #use upper bits 21-22 combined with modelSize for row index
 row=(hashValue & 0x00300000 >> 16) | (modelSize %16)
 #use lower 16 bits for the column index
 column=hashValue & 0x0000FFFF
 If (!bitValue[row][column])
  bitValue[row][column]=1;
  {return(0)}
 EndIf
 Else
  bitValue[row][column]==1; return(1)
 EndIf
EndFunction
```

1.3 Cholesky update

```
Function CholeskyInsert (model,varIndex){
 newModel=model.addVariable(varIndex);
 columnVector=dataMatrixSquaredLookup(varIndex);
 newMatrix=AddColumn(model.matrix,columnVector)
 newModel.matrix=GivensTriangularize(newMatrix);
 newModel.regressionParms=BackSubstitute(newModel.matrix)
 return(newModel)
EndFunction
```

1.4 Transitive reduction

```
edgeDistance[]=convertToNegativeLogs(edgeWeights)
edgeList.sortbyEdgeWeights()
Foreach edge in edgeList
 edgeDist=edgeDistance[edge.parent,edge.child]
 dist=findMinDistance(edge.parent,edge.child,edgeDist,edgeDistance)
 If(dist < minDist)
  edgeList.remove(edge)
 EndIf
EndForeach

Function findMinDistance(startNode,endNode,maxDist,edgeDistance)
 Priority Queue Q
 Q.push(endNode)
 While(!Q.empty())
  If(Q.top == startNode)BREAK #found path
  currentNode=Q.pop()
  pathDist=currentNode.pathDistance
  Foreach parentNode in currentNode.parentList
   parentDist=pathDist+edgeDistance[parentNode,currentNode]
   If(parentDist<maxDist)
    Q.Push(parentNode)
    parentNode.pathDistance=parentDist
   EndIf
  EndForeach
EndWhile
Return(Q.pop())
```
