# Supplementary material for: fastBMA: scalable network inference and transitive reduction
Source: Gigascience. 2017 Aug 22;6(10):1–10. doi: 10.1093/gigascience/gix078 (PMC5632288; doi:10.1093/gigascience/gix078)
Supplement: Response-to-Reviewer-Comments_Original-Submission.pdf [file gix078_Response-to-Reviewer-Comments_Original-Submission.pdf]

Author's Response To Reviewer Comments

We greatly appreciate the feedback from both reviewers and feel that the manuscript has been markedly improved with their feedback.

Reviewer 1
1) The authors claim that the transitive reduction based network post-processing method is a novel and important feature of their algorithm. Firstly, very similar techniques were previously used in many papers, some of which were cited by the authors in their manuscript. Therefore, I do not think it is appropriate to call it novel. Secondly, in the benchmarking studies, the transitive reduction method did not seem to improve the accuracy of the networks inferred by the fastBMA algorithm. If it does not improve the performance of fastBMA then why is it being packaged together with fastBMA and being presented as an important feature of the fastBMA algorithm?
The point is well taken that a reader might well construe "novel" to refer to overall transitive reduction approach based on the existence of a better paths, rather than the shortest path mapping approach which is the "novel" part of the implementation. We have removed the word "novel" to avoid confusion.

Response
While the methodology did not improve the sparse fastBMA networks, it did improve the LASSO networks which were less sparse and is a very fast and natural quantitative extension of the original method proposed by Wagner (who did not use edge weights). To our knowledge, no one has directly mapped the search for better paths to a shortest path problem. Therefore we included it as a module for those who wish to use this type of methodology for denser graphs generated by fastBMA on different datasets or from graphs generated using different methods. A few sentences have been added to the discussion to better explain this.

2) In the "Background" section (under "Findings") the authors cited many relevant research papers. However, in the regression based methods category the authors mostly cited their own work. I thinks the authors should cite other similar works in the same category, e.g. doi:10.1038/srep37140,http://d/bti487.

Response
We have added the first reference. The url to the second reference was mistyped and at first we could not identify it. However, we guessed that the reviewer meant doi:10.1093/bioinformatics/bti487. So, we added Rogers et al. as well.

3) It seems that the underlying principles of the fastBMA algorithm is written under the heading "Related work". This is confusing since "related work" typically refers to similar work by other researchers.

Response
This is a good point. We have removed the heading to avoid confusion.

4) The authors claimed that their algorithm can incorporate prior knowledge of the network

topology in the inference process. In the benchmarking studies they have shown how prior knowledge improve the performance of their algorithm. However, I did not find a description of how prior knowledge is incorporated in the core algorithm. A brief description of this process will help readers understand the algorithm in its entirety.

Response
The methodology starts with a list of prior probabilities (edge weights). When these are NOT provided, we use a uniform non-informative prior for the starting edge weights. We have added a few sentences to the methods to make this clearer in the Background section to clarify.

5) The benchmarking studies performed in this manuscript are not convincing. The authors did not compare the performance of their algorithm with some of the most well known methods such as GENIE3 (http://dx.doi.org/10.1371/jou and JUMP3), JUMP3 (10.1093/bioinformatics/btu863) which were shown to be significantly superior to algorithms such as ARACNE, MRNET, CLR etc. which were used to compare the performance of scanBMA whose performance was compared with the fastBMA algorithm in this manuscript. To gain a better understanding of where their algorithm stands in terms of accuracy, compared to the current state of the art, they should compare the performances of their algorithm with the current top performers.

Response
The point is well taken that we have only focused on computationally efficient methods such as LASSO which can scale to large numbers of genes and large numbers of samples and have not compared with other methods that may be suitable for smaller networks. While the original GENIE3 paper did not address the performance of on Dream4 time-series data, the follow-up paper on Jump3 did, allowing us to compare the results. We have added Table 2 to compare the results and relevant sentences to the Methodology, Results and Discussion sections.

6) The authors did not properly discuss the weaknesses of their algorithm, for instance in which scenarios their algorithm is not expected to perform well?

Response
We have added a few sentences to the discussion about the potential weaknesses of the approach – mainly the sampling of a small search space around an initial set of good models which is very fast and efficient, but may miss optimal solutions when there are many dissimilar models of similar quality -when more thorough sampling is possible and warranted.

Reviewer 2

1. For technical notes, the article sections should include only "Findings" and "Methods", which can then be broken down into subsections. While this has been done for a portion of the article, the article flow could be improved significantly to increase the clarity of the scientific content. Conclusions should also be moved into a subheading of "Findings", instead of falling after "Methods". Results should also be integrated into the "Findings" section.

Response

By our subheading “Methods” we did not mean protocols or workflows used to reproduce results which is what is meant by Gigascience section heading “Methods”. We meant methodology of the new technique which do belong in the Findings section. We have re-labeled the section fastBMA Methodology to clear up the confusion.

2. Would recommend placing "Related Work" in the background and integrating "Our Contributions", rather than including this as a separate section.

Response
We have removed the headings and re-organized the order of the text to improve the flow of the article.

3. Several references to the speed of fastBMA are made in the Background/Contributes/Related Work sections, without any supporting evidence or figures in those sections.
- Second paragraph of "Our Contributions" in 2 locations
- In "Estimating model posterior probabilities" and others, should indicate/explain what is meant by "faster C++ code" for fastBMA -- do the other applications use a different language? Less performant algorithms?

Response
Quite a bit of prototyping work went into the methodology that does not make it into the final manuscript. We now refer to this explicitly in the text so that the reader understands that this is not a major point that is established by the figures and tables. In the above paragraphs, the speed increases referred to are in figure 4A and discussed later in the methodology and the text has been adjusted to make this clear.
The “faster C++ code” is indeed confusing. ScanBMA uses R for the EM – fastBMA uses almost the same algorithm in C++ which is faster. The text has been adjusted to explicitly state this.

4. The implementation methods of fastBMA are also described in the "Our Contributions" section, prior to "Related Work"

Response
This is meant to be a précis of what was is new in the work, intended for those who may not read the subsequent sections carefully. There is a bit of repetition necessary in order to make this section self-contained.

5. Methods are written more like results (ie, "Algorithmic outline..."discusses the performance enhancements rather than just the approach) and discussion sections instead of being used as an explanation of implementation details and data sets-

Response
The optimization process involved prototyping with many different versions. While the details are really not essential to final results, we do feel they are of use to the reader who is considering whether to perform similar optimizations and who may want to know how varying each component roughly affects the speed. We have adjusted the text to clearly indicate when we are referring to internal benchmarks and prototyping rather than the major results reported in the

manuscript.

"Replacing the hash table" has similar issues, and also discusses "crashing a 56 GB machine" with minimal explanation (possibly out of memory? unclear how large of a dataset for this to occur).

Response
We have changed the text to make it clear that this is due to running out of memory. The conditions when this happens are described in the text – uninformative priors – large search windows. Exactly when this will happen is hard to predict beforehand because it depends on the number of models sampled (as described in the text) and not the size of the dataset per se.

- Most of the "Replacing the hash table" section appears to reference ScanBMA rather than fastBMA -- would focus on methods of fastBMA and how this improves on the prior work in the findings, instead of going into in-depth explanations in the methods
As noted by the reviewer, the fastBMA approach (probabilistic hash) and the benefits of using it are described in the final paragraphs of the section. We feel that it is necessary to describe the ScanBMA approach to understand why this is feasible, especially for readers less familiar with the inner workings of a hash table. We have added an introductory sentence to the text so that the reader knows the roadmap and skip to the section on fastBMA if desired.
- The end of this section states that fastBMA is much faster than using a full hash table, but no supporting data are provided (only a descriptionof the approach)

Response
This was done using internal prototyping with versions of fastBMA with and without the probabilistic hash and is now made clear in the text.

6. Figure 3 is never referenced in the text

Response
Figure 3 is now referenced in the text

7. The text in the section "Transitive reduction to eliminate redundant edges" is not entirely clear. While the purpose is in the title, the text does not necessarily support the title, nor offer any evidence (figures,data) to support the conclusions in the section

Response
This is a bad title which we have changed to “Transitive reduction: eliminating edges when there is a better indirect path.” We have changed the text to make it clearer that the core idea derives from Wagner and is an established methodology – only the implementation using shortest paths is new.

8. While the fastBMA results in Fig 4B cannot all be compared to ScanBMA since runs with equivalent data were not possible, the statement that all fastBMA lines are to the left of

ScanBMA should be better explained in the text, as the larger fastBMA data with (without priors) takes as long or longer than ScanBMA (agree these cannot be compared, but the text does not explain this as currently written). This may be clarified by splitting references to Fig 4A and 4B in the text, rather than only referencing "Figure 4". May also want to explain why running with priors takes substantially less time than running with priors on fastBMA.

Response
We have changed the reference to 4A and added a sentence to explain why using priors results in shorter running times.
9. More background on what informative priors were used from external datasets may be of benefit
This was already done in detail in the ScanBMA paper referenced. We really tried to focus on the performance aspects of fastBMA in this technical note.

10. For the 32 core cluster, was this multiple machines totaling 32 cores? Or a single 32 core node?

Response
Two nodes of 16 cores. This has been clarified in the text.

11. Some discussion as to why the AUC is better in Fig 4A for fastBMA 8 core compared to fastBMA 1 core would be warranted

Response
The AUC is not better and this is indicated by the identical height/Y coordinate of the points.

12. The OR parameter used for fastBMA in Figure 5 should be stated, to better compare results from the AUC and Precision-Recall curves

Response
This has been added.

13. Can reduce the number of times links to the software in the article are referenced (ie, the Docker images are noted in the abstract, contributes, and conclusion)

Response
We have removed the link in the conclusions to avoid multiple references in a single section. We have kept the links in the different sections as readers may only look at the abstract, or main body of the paper.

14. For DREAM4 data set, both 10-gene and 100-gene data are referenced in the "Datasets" section, but not indicated which was used in the results/figures

Response
Actually all the datasets were merged and used to calculate AUC. This is now explicitly stated in the text.

15. A prior ScanBMA article appears to have used all 3556 variables in the Yeast data set (http://bmcsystbiol.biomedcent) -- any reason that ScanBMA was only run with 100 variables+prior here, instead of including the 3556 without prior?

Response
The previous work on the yeast dataset was restricted to using informative priors. Furthermore it only used an odds ratio of 100 and only assayed the effectiveness on 20 genes (while using all 3556 variables). Larger odds ratios and using the full 3556 genes just takes too long especially for 5 runs. A sentence has been added to the Results to better explain this.

16. Explanation of the software environment setup and its impact on performance/run time should be included -- were all tools installed on a single virtual machine? Running the same OS? Were they run within Docker containers? Any potential performance changes due to the use of shared/virtual hardware? Were the applications run a single time, or were they run multiple times to determine if there was any variability between runs based on potential storage/network capacity within the shared environment? Were data sets stored locally on within the instance?

Response
They were run 5 times on the same OS/instance and averaged. We had error bars originally but they were not visible because of the low variability. Containers were not used. This is now explained in the text and figure legend.
