# Supplementary material for: fastBMA: scalable network inference and transitive reduction
Source: Gigascience. 2017 Aug 22;6(10):1–10. doi: 10.1093/gigascience/gix078 (PMC5632288; doi:10.1093/gigascience/gix078)
Supplement: Response-to-Reviewer-Comments_Revision-1.pdf [file gix078_Response-to-Reviewer-Comments_Revision-1.pdf]

Author's Response To Reviewer Comments

We have included the priors data as supplemental materials added the following text describing the priors.

Specifically, we applied a supervised learning approach to a training dataset consisting of regulator-gene pairs and various attributes assembled from diverse gene expression data, genome-wide binding data, protein-protein interactions, GO terms and prior knowledge from the literature. We computed predicted probabilities of regulatory relationships using this supervised learning approach and these predicted probabilities were used as priors in the regression step. These priors are available in the lopriors.tsv file in the supplemental materials.
