# Supplementary material for: fastBMA: scalable network inference and transitive reduction
Source: Gigascience. 2017 Aug 22;6(10):1–10. doi: 10.1093/gigascience/gix078 (PMC5632288; doi:10.1093/gigascience/gix078)
Supplement: Response-to-Reviewer-Comments_Revision-2.pdf [file gix078_Response-to-Reviewer-Comments_Revision-2.pdf]

Author's Response To Reviewer Comments

The urls in the main body of the manuscript have been replaced by citations. The main github citation has been changed to the Gigascience Database citation for the supporting data.

I have added the line referring to the Gigascience Database in the available materials and also edited the citation to include the url.
