# Supplementary material for: fastBMA: scalable network inference and transitive reduction
Source: Gigascience. 2017 Aug 22;6(10):1–10. doi: 10.1093/gigascience/gix078 (PMC5632288; doi:10.1093/gigascience/gix078)
Supplement: Reviewer-1-Report-(Original-Submission).pdf [file gix078_Reviewer-1-Report-(Original-Submission).pdf]

Reviewer Report

Title: fastBMA: Scalable Network Inference and Transitive Reduction

Version: Original Submission **Date:** 3/1/2017

Reviewer name: Tapesh Santra

Reviewer Comments to Author:

The authors of the manuscript titled: "fastBMA: Scalable Network Inference and Transitive Reduction" have developed a fast and scalable gene regulatory network reconstruction algorithm which is a faster and more accurate version of their previous algorithm scanBMA. It also features a network post-processing method based on transitive reduction of graphs. Below are my comments on this manuscript.In general the manuscript is relevant to current research, especially in the field of systems biology and biostatistics. It is well written and clearly understandable. It is a welcome addition to the arsenal of scalable algorithms for gene regulatory network inference. However, I think the paper can be improved significantly by addressing the following comments.1) The authors claim that the transitive reduction based network post-processing method is a novel and important feature of their algorithm. Firstly, very similar techniques were previously used in many papers, some of which were cited by the authors in their manuscript. Therefore, I do not think it is appropriate to call it novel. Secondly, in the benchmarking studies, the transitive reduction method did not seem to improve the accuracy of the networks inferred by the fastBMA algorithm. If it does not improve the performance of fastBMA then why is it being packaged together with fastBMA and being presented as an important feature of the fastBMA algorithm?2) In the "Background" section (under "Findings") the authors cited many relevant research papers. However, in the regression based methods category the authors mostly cited their own work. I thinks the authors should cite other similar works in the same category, e.g. doi:10.1038/srep37140, http://dx.doi.org/10.1039/C4MB00053F,https://doi.org/10.1093/bioinformatics/bti487.3) It seems that the underlying principles of the fastBMA algorithm is written under the heading "Related work". This is confusing since "related work" typically refers to similar work by other researchers.4) The authors claimed that their algorithm can incorporate prior knowledge of the network topology in the inference process. In the benchmarking studies they have shown how prior knowledge improve the performance of their algorithm. However, I did not find a description of how prior knowledge is incorporated in the core algorithm. A brief description of this process will help readers understand the algorithm in its entirety.5) The benchmarking studies performed in this manuscript are not convincing. The authors did not compare the performance of their algorithm with some of the most well known methods such as GENIE3 (http://dx.doi.org/10.1371/journal.pone.0012776 and JUMP3), JUMP3 (10.1093/bioinformatics/btu863) which were shown to be significantly superior to algorithms such as ARACNE, MRNET, CLR etc. which were used to compare the performance of scanBMA whose performance was compared with the fastBMA algorithm in this manuscript. To gain a better understanding of where their algorithm stands in terms of accuracy, compared to the current state of the art, they should compare the performances of their algorithm with the current top performers.6)

The authors did not properly discuss the weaknesses of their algorithm, for instance in which scenarios their algorithm is not expected to perform well?

Level of Interest

Please indicate how interesting you found the manuscript: An article of importance in its field

Quality of Written English

Please indicate the quality of language in the manuscript: Acceptable

Declaration of Competing Interests

Please complete a declaration of competing interests, considering the following questions:

- Have you in the past five years received reimbursements, fees, funding, or salary from an organisation that may in any way gain or lose financially from the publication of this manuscript, either now or in the future?
- Do you hold any stocks or shares in an organisation that may in any way gain or lose financially from the publication of this manuscript, either now or in the future?
- Do you hold or are you currently applying for any patents relating to the content of the manuscript?
- Have you received reimbursements, fees, funding, or salary from an organization that holds or has applied for patents relating to the content of the manuscript?
- Do you have any other financial competing interests?
- Do you have any non-financial competing interests in relation to this paper?

If you can answer no to all of the above, write 'I declare that I have no competing interests' below. If your reply is yes to any, please give details below.

The reviewer declares no competing interest

I agree to the open peer review policy of the journal. I understand that my name will be included on my report to the authors and, if the manuscript is accepted for publication, my named report including any attachments I upload will be posted on the website along with the authors' responses. I agree for my report to be made available under an Open Access Creative Commons CC-BY license (http://creativecommons.org/licenses/by/4.0/). I understand that any comments which I do not wish to be included in my named report can be included as confidential comments to the editors, which will not be published.

I agree to the open peer review policy of the journal
