# Supplementary material for: fastBMA: scalable network inference and transitive reduction
Source: Gigascience. 2017 Aug 22;6(10):1–10. doi: 10.1093/gigascience/gix078 (PMC5632288; doi:10.1093/gigascience/gix078)
Supplement: Reviewer-1-Report-(Revision-1).pdf [file gix078_Reviewer-1-Report-(Revision-1).pdf]

Reviewer Report

Title: fastBMA: Scalable Network Inference and Transitive Reduction

Version: Revision 1 **Date:** 7/1/2017

Reviewer name: Tapesh Santra

Reviewer Comments to Author:

In general, the revised manuscript has improved compared to the initial submission. I have only on minor comment.
In line 35, page 26 the authors cited an earlier paper (ref: 12 in the main manuscript) from their own group as a source of external data that was used as prior knowledge for inferring the yest gene regulatory network. However, upon reading ref. 12 it was not immediately clear how this external data was collected and from where. The authors should clearly describe the sources of prior knowledge (that was used for yeast GRN reconstruction) in their manuscript. They should also provide the prior network in an easily accessible format as part of the supplementary material, this will enable readers to reproduce their results.

Level of Interest

Please indicate how interesting you found the manuscript: An article of importance in its field

Quality of Written English

Please indicate the quality of language in the manuscript: Acceptable

Declaration of Competing Interests

Please complete a declaration of competing interests, considering the following questions:

- Have you in the past five years received reimbursements, fees, funding, or salary from an organisation that may in any way gain or lose financially from the publication of this manuscript, either now or in the future?
- Do you hold any stocks or shares in an organisation that may in any way gain or lose financially from the publication of this manuscript, either now or in the future?
- Do you hold or are you currently applying for any patents relating to the content of the manuscript?
- Have you received reimbursements, fees, funding, or salary from an organization that holds or has applied for patents relating to the content of the manuscript?
- Do you have any other financial competing interests?
- Do you have any non-financial competing interests in relation to this paper?

If you can answer no to all of the above, write 'I declare that I have no competing interests' below. If your reply is yes to any, please give details below.

I declare no competing interest

I agree to the open peer review policy of the journal. I understand that my name will be included on my report to the authors and, if the manuscript is accepted for publication, my named report including any attachments I upload will be posted on the website along with the authors' responses. I agree for my report to be made available under an Open Access Creative Commons CC-BY license (http://creativecommons.org/licenses/by/4.0/). I understand that any comments which I do not wish to be included in my named report can be included as confidential comments to the editors, which will not be published.

I agree to the open peer review policy of the journal
