# Supplementary material for: fastBMA: scalable network inference and transitive reduction
Source: Gigascience. 2017 Aug 22;6(10):1–10. doi: 10.1093/gigascience/gix078 (PMC5632288; doi:10.1093/gigascience/gix078)
Supplement: Reviewer-2-Report-(Original-Submission).pdf [file gix078_Reviewer-2-Report-(Original-Submission).pdf]

Reviewer Report

Title: fastBMA: Scalable Network Inference and Transitive Reduction

Version: Original Submission **Date:** 3/12/2017

Reviewer name: Wade Schulz

Reviewer Comments to Author:

In the article "fastBMA: Scalable Network Inference and Transitive Reduction", the authors present an improved tool, fastBMA, as an extension of their prior work on the inference of genetic networks. Most comments below are related to the article text and writing style, rather than any major concerns related to the scientific results. However, there should be significant adjustment to the text to improve the scientific clarity of the findings (ie, not all figures in the article are referenced in the text, article does not follow style guidelines, etc).1. For technical notes, the article sections should include only "Findings" and "Methods", which can then be broken down into subsections. While this has been done for a portion of the article, the article flow could be improved significantly to increase the clarity of the scientific content. Conclusions should also be moved into a subheading of "Findings", instead of falling after "Methods". Results should also be integrated into the "Findings" section.2. Would recommend placing "Related Work" in the background and integrating "Our Contributions", rather than including this as a separate section.3. Several references to the speed of fastBMA are made in the Background/Contributes/Related Work sections, without any supporting evidence or figures in those sections.- Second paragraph of "Our Contributions" in 2 locations- In "Estimating model posterior probabilities" and others, should indicate/explain what is meant by "faster C++ code" for fastBMA -- do the other applications use a different language? Less performant algorithms?4. The implementation methods of fastBMA are also described in the "Our Contributions" section, prior to "Related Work"5. Methods are written more like results (ie, "Algorithmic outline..." discusses the performance enhancements rather than just the approach) and discussion sections instead of being used as an explanation of implementation details and data sets- "Replacing the hash table" has similar issues, and also discusses "crashing a 56 GB machine" with minimal explanation (possibly out of memory? unclear how large of a dataset for this to occur).- Most of the "Replacing the hash table" section appears to reference ScanBMA rather than fastBMA -- would focus on methods of fastBMA and how this improves on the prior work in the findings, instead of going into in-depth explanations in the methods- The end of this section states that fastBMA is much faster than using a full hash table, but no supporting data are provided (only a description of the approach)6. Figure 3 is never referenced in the text7. The text in the section "Transitive reduction to eliminate redundant edges" is not entirely clear. While the purpose is in the title, the text does not necessarily support the title, nor offer any evidence (figures, data) to support the conclusions in the section8. While the fastBMA results in Fig 4B cannot all be compared to ScanBMA since runs with equivalent data were not possible, the statement that all fastBMA lines are to the left of ScanBMA should be better explained in the text, as the larger fastBMA data with (without priors) takes as long or longer than ScanBMA (agree these cannot be compared, but the text does not explain this as currently written). This may be clarified by splitting references to Fig 4A and 4B in the text, rather than

only referencing "Figure 4". May also want to explain why running with priors takes substantially less time than running with priors on fastBMA.9. More background on what informative priors were used from external data sets may be of benefit10. For the 32 core cluster, was this multiple machines totaling 32 cores? Or a single 32 core node?11. Some discussion as to why the AUC is better in Fig 4A for fastBMA 8 core compared to fastBMA 1 core would be warranted12. The OR parameter used for fastBMA in Figure 5 should be stated, to better compare results from the AUC and Precision-Recall curves13. Can reduce the number of times links to the software in the article are referenced (ie, the Docker images are noted in the abstract, contributes, and conclusion)14. For DREAM4 data set, both 10-gene and 100-gene data are referenced in the "Datasets" section, but not indicated which was used in the results/figures15. A prior ScanBMA article appears to have used all 3556 variables in the Yeast data set (http://bmcsystbiol.biomedcentral.com/articles/10.1186/1752-0509-8-47) -- any reason that ScanBMA was only run with 100 variables+prior here, instead of including the 3556 without prior?16. Explanation of the software environment setup and its impact on performance/run time should be included -- were all tools installed on a single virtual machine? Running the same OS? Were they run within Docker containers? Any potential performance changes due to the use of shared/virtual hardware? Were the applications run a single time, or were they run multiple times to determine if there was any variability between runs based on potential storage/network capacity within the shared environment? Were data sets stored locally on within the instance?

Level of Interest

Please indicate how interesting you found the manuscript: An article of importance in its field

Quality of Written English

Please indicate the quality of language in the manuscript: Acceptable

Declaration of Competing Interests

Please complete a declaration of competing interests, considering the following questions:

- Have you in the past five years received reimbursements, fees, funding, or salary from an organisation that may in any way gain or lose financially from the publication of this manuscript, either now or in the future?
- Do you hold any stocks or shares in an organisation that may in any way gain or lose financially from the publication of this manuscript, either now or in the future?
- Do you hold or are you currently applying for any patents relating to the content of the manuscript?
- Have you received reimbursements, fees, funding, or salary from an organization that holds or has applied for patents relating to the content of the manuscript?
- Do you have any other financial competing interests?
- Do you have any non-financial competing interests in relation to this paper?

If you can answer no to all of the above, write 'I declare that I have no competing interests' below. If your reply is yes to any, please give details below.

I declare that I have no competing interests

I agree to the open peer review policy of the journal. I understand that my name will be included on my report to the authors and, if the manuscript is accepted for publication, my named report including any attachments I upload will be posted on the website along with the authors' responses. I agree for my report to be made available under an Open Access Creative Commons CC-BY license (http://creativecommons.org/licenses/by/4.0/). I understand that any comments which I do not wish to be included in my named report can be included as confidential comments to the editors, which will not be published.

I agree to the open peer review policy of the journal
